# Fatal Cryptococcal Meningitis in a Patient With Chronic Lymphocytic Leukemia Treated With Ibrutinib

**DOI:** 10.7759/cureus.37891

**Published:** 2023-04-20

**Authors:** Hari Oumayma, El Mehdi Mahtat, Hawa Moussa Bouh, Hicham Elmaaroufi, Kamal Doghmi

**Affiliations:** 1 Hematology Department, Military Hospital, Rabat, MAR

**Keywords:** chronic lymphocytic leukemia, cryptococcus neoformans, meningitis, cryptococcus neoformans (c. neoformans), risk factor, chronic lymphocytic leukemia (cll), ibrutinib

## Abstract

According to the latest World Health Organization classification published in 2022, chronic lymphocytic leukemia (CLL) is classified as a low-grade proliferation of clonal B-cells. The Bruton tyrosine kinase (BTK) pathway plays a crucial role in B-cell receptor signaling. Ibrutinib, the first irreversible BTK inhibitor, has been shown to improve the survival of CLL patients with lower toxicity than traditional chemotherapy.

Cryptococcosis is an invasive fungal infection that primarily affects individuals with compromised immune systems. We present a case of a 69-year-old male with relapsed CLL who received treatment with ibrutinib and subsequently developed meningeal cryptococcosis, presenting with seizures and fever. A physical exam showed bilateral hypoacusis, but no focal deficits. Cerebral imaging was normal and laboratory results showed a low gamma globulin level and leucopenia with lymphopenia but without neutropenia. The cerebrospinal fluid profile was not inflammatory, opening pressure was normal, the classic India ink test was positive, and fungal cultures grew *Cryptococcus neoformans*. To complete investigations, HIV testing was negative, and sinus and chest tomography scans showed no anomalies. Treatment consisted of discontinuing ibrutinib and administering anti-fungal therapy with liposomal amphotericin (4 mg/kg/day) in combination with flucytosine (25 mg/kg/day). However, the patient's neurological status declined, and he passed away.

This case highlights the potential risk of developing opportunistic infections such as cryptococcal meningitis in CLL patients treated with ibrutinib. It is crucial to consider the patient's immune status when administering ibrutinib and to closely monitor for signs of infection.

## Introduction

Chronic lymphocytic leukemia (CLL) accounts for approximately a quarter of all cases of leukemia, and is the most common type of leukemia among adults in Western countries [1].

The Bruton tyrosine kinase (BTK) pathway plays a pivotal role in promoting the proliferation and survival of B cells by mediating B-cell receptor signaling [2]. Ibrutinib, the first irreversible BTK inhibitor, has been shown to enhance the survival of CLL patients when used as a primary therapy [3] and in relapsed or refractory settings with lower toxicity compared to traditional chemotherapy [2]. Cryptococcosis is the third most prevalent invasive fungal infection [4], typically affecting individuals with the human immunodeficiency virus (HIV-1) [5]. *Cryptococcus* often occurs after conditioning chemotherapy and prolonged corticosteroid treatment [5].

Here, we report a case of a patient with a relapse of his CLL treated with ibrutinib, which was complicated by a fatal infection with cryptococcal meningitis.

## Case presentation

A 69-year-old Moroccan male patient was diagnosed with Binet stage A CLL. Fluorescence in situ hybridization (FISH) showed an 11q deletion without a TP53 mutation. At the time of diagnosis, the patient did not meet the criteria for treatment. Two years after diagnosis, he developed progressive disease and B symptoms (night sweats and fatigue). He then began standard treatment with fludarabine, cyclophosphamide, and rituximab (FCR) for six cycles, which resulted in complete remission. Three years later, the patient experienced a relapse of the disease and was treated with FCR once again, achieving complete remission after six cycles. He relapsed four months later. He was then started on ibrutinib at a dose of 420 mg/day in combination with trimethoprim-sulfamethoxazole to prevent *Pneumocystis jirovecii* infections and viral prophylaxis with valacyclovir.

After seven weeks of ibrutinib treatment, the patient presented to the emergency department with seizures. Upon admission, the patient was stable but had a fever of 39.5°C. Neurological examination showed bilateral hypoacusis, but no focal deficits. Cerebral imaging by tomography did not reveal any acute ischemic or hemorrhagic events.

**Figure 1:**
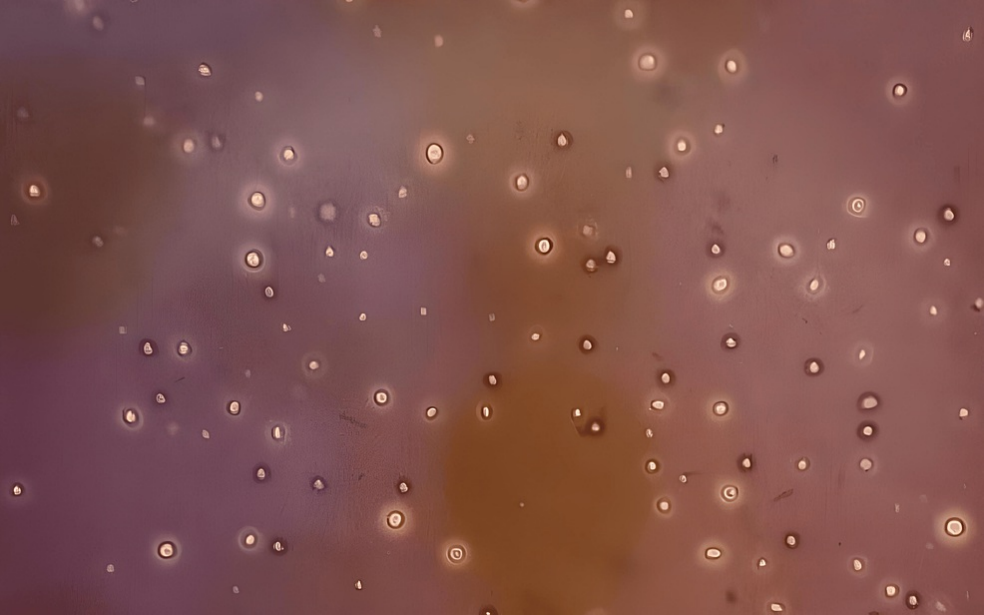
India ink preparation showing Cryptococcus neoformans in cerebrospinal fluid.

Blood investigations showed leucopenia at 3.6 giga/L, lymphopenia at 0.8 giga/L without neutropenia, and a low gamma globulin level of 0.32 g/L (Table 1). Cerebrospinal fluid (CSF) analysis showed four nucleated cells/mL, four red blood cells (RBCs)/mL, glucose at 57 mg/dL (glucose ratio not available), and protein at 43 mg/dL (Table 1), and opening pressure was normal. The classic India ink test was positive (Figure 1), and the fungal culture grew *Cryptococcus neoformans*. HIV testing was negative, and the CD4 lymphocytes were not quantified. Sinus and chest computed tomography (CT) scans showed no anomalies.

**Table 1:** Blood and CSF analysis findings. CSF: cerebrospinal fluid; PN: polynuclear neutrophils; RBC: red blood cells; WBC: white blood cells.

	Results	Normal range
Blood investigations		
WBC	3.6 giga/L	5-10 giga/L
Lymphocytes	0.8 giga/L	1- 4.8 giga/L
PN	2.5 giga/L	2.5- 6 giga/L
Gamma globulin level	0.32 g/L	7- 16 g/L
CSF analysis		
WBC	4 cells/mL	<5 cells/mL
RBC	4 cells/mL	0 cells/mL
Protein	43 mg/dL	15- 60 mg/dL
Glucose	57 mg/dL	50-80 mg/dL

The patient initiated antifungal therapy with liposomal amphotericin (4 mg/kg/day) in combination with flucytosine (25 mg/kg/day), and ibrutinib was stopped. The patient presented with persistent fever and thrombocytopenia. Despite repeated investigations, including aerobic and anaerobic cultures, body scanning, and fungal cultures, no infectious etiology was identified. Additionally, a CSF puncture could not be performed due to thrombopenia. Empiric antibiotic therapy with tazocillin and vancomycin was initiated along with antifungal therapy, but apyrexia was not achieved. On day 16, the patient presented a refractory status epilepticus and experienced a decline in neurological status. Cerebral imaging was normal. The patient was transferred to the intensive care unit but unfortunately passed away.

## Discussion

In patients with CLL, almost one-third of deaths are due to infectious complications [6]. This high predisposition to infections is attributed to defects in humoral immunity and in neutrophil and natural killer (NK) cell functions, which are exacerbated by immunochemotherapy [7]. The main pathogenic agents are encapsulated bacteria (*Streptococcus pneumoniae* and *Haemophilus influenzae*), *Staphylococcus aureus*, *Pseudomonas aeruginosa*, *Escherichia coli*, and *Klebsiella pneumoniae* [8]. Viral infections, such as herpes simplex and zoster, are more frequent in the advanced stages of the disease [8]. Little is known about the incidence of invasive fungal infections in CLL.

Early clinical trials showed a good safety profile for ibrutinib, with the most common side effects being grade 1-2 diarrhea, edema, fatigue, and rash. These side effects are generally transient and often do not require therapeutic intervention or interruption of treatment [9-12]. In a recent study involving patients with CLL and non-Hodgkin's lymphomas receiving ibrutinib treatment, nine patients developed cryptococcal infections, with six of these patients experiencing central nervous system involvement [12]. Furthermore, numerous authors have documented isolated occurrences of cryptococcal infections in patients with CLL who are undergoing treatment with ibrutinib. We have summarized our case report and similar documented cases in Tables 2, 3.

**Table 2:** Clinical features, management, and outcomes of different case reports. BOOP: bronchiolitis obliterans organizing pneumonia; CKD: chronic kidney disease; CNS: central nervous system.

	Age	sex	Cryptococcal endemic in the region of residence	Comorbidities	Prior treatment	Clinical presentation	Time to start ibrutinib	Localization	Management	Outcomes
Case 1 [13]	68	Female	Non specified	Diabetes, hypertension, and dyslipidemia	Chlorambucil and corticosteroids	Fever and transient lower lip numbness	3 months	CNS and lungs	Liposomal amphotericin and flucytosine for 2 weeks; interruption of ibrutinib	Recovering; reintroduction of ibrutinib (140 mg daily) associated with fluconazole
Case 2 [14]	66	Male	Non specified	Diabetes, hypertension, and dyslipidemia	Hematopoietic stem cell transplant; corticosteroids and chlorambucil; rituximab and bendamustine	Nausea and vomiting; confusion and fever	6 months	CNS and sinus	Liposomal amphotericin and flucytosine for 2 weeks; interruption of ibrutinib	Recovering; reintroduction of ibrutinib (140 mg daily) associated with fluconazole
Case 3 [15]	54	Male	Non specified	Obesity, diabetes, BOOP, and CKD	Cyclophosphamide, fludarabine, and rituximab	Fever and hypoxia	4 weeks	CNS, blood, and lungs	Liposomal amphotericin for 28 days and flucytosine for 14 days; interruption of ibrutinib	Respiratory failure and death
Case 4 [16]	83	Male	Non specified	Non specified	Cyclophosphamide, fludarabine and rituximab	Hypoxia and sepsis	Non specified	CNS and blood	Liposomal amphotericin and flucytosine for 2 weeks; interruption of ibrutinib	Recovering; reintroduction of ibrutinib (dose reduction to 50%) associated with fluconazole
Our case	69	Male	Absence	None	Cyclophosphamide, fludarabine and rituximab	Fever and seizures	7 weeks	CNS	Liposomal amphotericin and flucytosine until death (16 days); interruption of ibrutinib	Decline of neurologic status and death

**Table 3:** Biologic findings in different reported cases. Abnormal values are in bold. (): normal ranges; BAL: bronchoalveolar lavage; *C. neoformans*: *Cryptococcus neoformans*; CSF: cerebrospinal fluid; CNS: central nervous system; HIV: human immunodeficiency virus; PN: polynuclear neutrophils; Lymph: lymphocytes; RBC: red blood cells; WBC: white blood cells.

	Profile of immunodeficiency				Site of diagnosis	Biologic findings in CSF		
	PN (2.5-6 giga/L)	Lymph (1-4.8 giga/L)	Gamma globulin level (7-16 g/L)	HIV status		Cytology (WBC < 5/mL; RBC = 0/mL)	Biochemistry (glucose: 50-80 mg/dL; protein: 15-60 mg/dL)	Mycology
Case 1 [13]	4.9	13.26	0.56	Non-specified	Blood	1 WBC/mL; RBC: non-specified	Non-specified	Cryptococcal antigen negative
Case 2 [14]	Non-specified	0.5	Low	Non-specified	CSF	123 WBC/mL; 17 RBC/mL	Glucose: 62 mg/dL; protein: 147 mg/dL	Cryptococcal antigen positive
Case 3 [15]	Non-specified	Non-specified	Non-specified	Non-specified	CSF, blood, and BAL	4 WBC/mL; 6 RBC/mL	Glucose: 157 mg/dL; protein: 30 mg/dL	Negative CSF cryptococcal antigen; fungal culture: *C. neoformans*
Case 4 [16]	2.7	0.19	0.45	Negative	Blood, CSF	6 WBC/ml; RBC: non-specified	Non-specified	Fungal culture: *C. neoformans*
Our case	2.5	0.88	0.32	Negative	CSF	4 WBC/mL; 4 RBC/mL	Glucose: 57 mg/dL; protein: 43 mg/dL	India ink test positive; fungal culture: *C. neoformans*

In our analysis of these cases, disseminated cryptococcosis (defined as an infection in two or more sites) was present in four patients. None of the reported cases presented with neutropenia; however, all patients had low gamma globulin levels when available. Cryptococcosis was diagnosed at a median of 65 days after initiation of ibrutinib therapy, with *C. neoformans* being the causative agent in all cases. Concerning clinical manifestations, all patients exhibited fever, and three presented with neurological symptoms, including transient lower lip numbness, confusion, and seizures. The diagnostic site was variable, with some cases being diagnosed based on blood culture, CSF, and bronchoalveolar lavage (BAL). Only one patient had a manifest inflammatory CSF profile with a high rate of white blood cells.

All patients received treatment with liposomal amphotericin B 4 mg/kg/day with flucytosine 25 mg/kg/day and discontinued ibrutinib, but two patients died at two and four weeks after diagnosis; patients successfully treated were maintained on secondary prophylaxis with fluconazole while ibrutinib was restarted at a reduced dose given the potential hepatic cytochrome P450 interaction with fluconazole.

The diffusion of ibrutinib across the blood‐brain barrier is well-established. Murine models show that ibrutinib can pass through the blood‐brain barrier in 0.29 hours and achieve a concentration in the CNS equivalent to plasma levels [17]. This may lead to the inhibition of CNS macrophages, increasing the risk of fungal dissemination.

Furthermore, ibrutinib can alter caspase recruitment domain homologs (CARD9 and CARD11), which play a crucial role in regulating the response of macrophages and microglial cells against the dissemination of fungal infections within the CNS [18].

## Conclusions

We describe a case of fatal cryptococcal meningitis seven weeks after starting treatment for relapsing CLL with ibrutinib. The precise relationship between ibrutinib and *Cryptococcus* infection remains incompletely understood. However, previous reports suggest that ibrutinib may heighten susceptibility to cryptococcal meningitis. In light of these findings, clinicians should weigh the potential benefits of antifungal prophylaxis for individuals at elevated risk of acquiring *Cryptococcus* infections.
